# Supplementary material for: Chromothripsis during telomere crisis is independent of NHEJ, and consistent with a replicative origin
Source: Genome Res. 2019 May;29(5):737–49. doi: 10.1101/gr.240705.118 (PMC6499312; doi:10.1101/gr.240705.118)
Supplement: Supplemental Material [file supp_gr.240705.118_Supplemental_file_1.zip › contigs/annotated_contigs/DB79/contig.2.DB79_length_641_mean_cov_16.6146645866.docx]

**DB79_length_641_mean_cov_16.6146645866**

```
CTCGTGTTTTGACCCATATCCTCAGGATTTTGATCTTCTTTCAGTTAATCTCTTTATGTTAATTCTTTCTTTTTTCTCTTTATGTTAAT
 >chr9:118642332-118642605 - E=2e-146
TCTTAATGCAACATCAATGAAACTTATAGTTATAAATCTTTTGGGAAAAGTGGTTTGCACTTCCACTGAATTACAGATCTGAGATATGG

GTGAATCTTTAAAGTACTGGTTCAATATCCTACCTGATCTTGAAAGAACTTGAGGTTTTTAGCTGAAGATCAGAGTGTCTGCTGTTCCA

GAGA|GC|TGATGAGTTAGAATCACCTGGCAAACTTTTAAGATTATTGGACCCTGCCTCAGATGTTCCCCAAGTTAAACTCTCTGAAAA
     >chr9:123541667-123542037 - E=6e-208
TAGGGACCTGCCTGTGTGTTTTGATAAAGTTGTATCACATGGCCATTGAGAACCAACCAAGTCCTACCCCTCTTTTTATACAGAAGGAA

ACAGTCTAAATAGCAAAGTGACAATAAATACAGTCTTTATTTCAGTATGCTATCATAGTTTTCCCAGGTTAAAATTCATACTCTAGCTC

TTTCATAAGTGATGGCTANTTTTCCATTGTCTCAAACAGTGGTTCTTGACTGAAGGTAGAATTGTGGCTGGCTTTAGTCATTTCAGCTT

TTTAAAAACTGGTCATTTGG
```
